# Comparable outcomes between immune-tolerant and active phases in noncirrhotic chronic hepatitis B: a meta-analysis

**DOI:** 10.1097/HC9.0000000000000011

**Published:** 2023-01-18

**Authors:** Han Ah Lee, Seung Up Kim, Yeon Seok Seo, Sang Hoon Ahn, Chai Hong Rim

**Affiliations:** 1 Department of Internal Medicine, College of Medicine, Ewha Womans University, Seoul, Korea; 2 Department of Internal Medicine, Yonsei University College of Medicine, Seoul, Korea; 3 Yonsei Liver Center, Severance Hospital, Seoul, Korea; 4 Departments of Internal Medicine, Korea University College of Medicine, Seoul, Korea; 5 Department of Radiation Oncology, Korea University College of Medicine, Seoul, Korea; 6 Department of Radiation Oncology, Korea University Ansan Hospital, Gyeonggi-do, Korea

## Abstract

Antiviral therapy is not indicated for patients with chronic hepatitis B (CHB) in the immune-tolerant (IT) phase. We compared the outcomes between the untreated IT phase and the treated immune-active (IA) phase in noncirrhotic HBeAg-positive CHB patients.

**Methods:**

We systematically searched 4 databases, including PubMed, Medline, Embase, and Cochrane, until August 2021. The pooled incidence rates of HCC and mortality in the IT and IA cohorts and phase change in the IT cohort were investigated. Studies that included patients with liver cirrhosis were excluded.

**Results:**

Thirteen studies involving 11,903 patients were included. The overall median of the median follow-up period was 62.4 months. The pooled 5-year and 10-year incidence rates of HCC were statistically similar between the IT and IA cohorts (1.1%, 95% CI: 0.4%–2.8% vs. 1.1%, 95% CI: 0.5%–2.3%, and 2.7%, 95% CI: 1.0%–7.3% vs. 3.6%, 95% CI: 2.4%–5.5%, respectively, all *p*>0.05). The pooled 5-year odds ratio of HCC between IT and IA cohorts was 1.05 (95% CI: 0.32–3.45; *p*=0.941). The pooled 5-year incidence rate of mortality was statistically similar between the IT and IA cohorts (1.9%, 95% CI: 1.1%–3.4% vs. 1.0%, 95% CI: 0.3%–2.9%, *p*=0.285). Finally, the pooled 5-year incidence rate of phase change in the IT cohort was 36.1% (95% CI: 29.5%–43.2%).

**Conclusion:**

The pooled incidence rates of HCC and mortality were comparable between the untreated IT and the treated IA phases in noncirrhotic HBeAg-positive CHB patients.

## INTRODUCTION

HBV infection is a significant global health problem, affecting ~350 million people worldwide.^1^,^2^ It has been known that high serum HBV-DNA level, a representative marker of active HBV replication in hepatocytes, is associated with an increased risk of severe complications such as liver cirrhosis and HCC.^3^,^4^ Therefore, continuous suppression of viral replication using potent antiviral therapy (AVT) has been a key therapeutic strategy to improve long-term prognosis in patients with chronic hepatitis B (CHB).^5^

According to international guidelines, AVT is indicated in the immune-active (IA) phase in patients with HBeAg-positive CHB.^5^–^8^ In contrast, the immune-tolerant (IT) phase, which is characterized by a high serum HBV-DNA level and normal alanine aminotransferase (ALT) level, is not indicative of AVT, because it is known that the IT phase has a minimal liver injury in histology and thus has a negligible risk of liver disease progression.^9^,^10^

However, a considerable risk of HCC development in patients with untreated IT phase, ranging from 6.2% to 12.7% at 10 years has been reported.^11^,^12^ A study by Kim et al^11^ showed that the 10-year cumulative incidence rate of HCC and death/transplantation was significantly higher in patients in the untreated IT phase than that in patients in the IA phase treated with AVT. However, due to insufficient histological information and potential bias owing to insufficient exclusion of patients with a higher probability of advanced liver fibrosis, particularly in the IT phase, this finding should be carefully interpreted.

In this meta-analysis, we compared the pooled incidence rates of HCC and mortality between the untreated IT phase and treated IA phase in noncirrhotic HBeAg-positive CHB patients and that of phase change in patients in the IT phase.

## METHODS

### Search and selection of eligible studies

This study was designed to investigate the pooled incidence rates of HCC and mortality in the untreated IT phase (IT cohort) and IA phase treated with AVT (IA cohort) in noncirrhotic HBeAg-positive CHB patients. All studies in this meta-analysis performed at least 2 tests during 6–12 months of observation period to define IT phase. We adhered to PRISMA in conduction and referenced the Cochrane Handbook version 6.2 for methodological regard.^13^,^14^ Eligible studies should meet the following criteria: (1) clinical studies including IT or IA cohorts; (2) at least 10 patients should be evaluated; and (3) cumulative incidence rates of HCC or mortality should be provided. Studies including patients with both CHB and other liver diseases such as alcoholic liver disease and hepatitis C virus infection were excluded.

We excluded studies that recruited patients with clinically or pathologically diagnosed liver cirrhosis for several reasons. First, we attempted to remove the influence of liver cirrhosis on the risk of developing HCC or mortality for an accurate comparison between different CHB phases.^15^ Second, without excluding patients with liver cirrhosis, it might be plausible that patients with early compensated liver cirrhosis could be inappropriately allocated to the IT phase group.

We systematically searched 4 databases including PubMed, Medline, Embase, and Cochrane Library, for publications until August 5, 2021. The search strategy, including search terms according to databases, is shown in Supplement Note 1, http://links.lww.com/HC9/A37. The following criteria were prioritized for the studies from the same institution: (1) comparative study (eg, studies with both IT and IA cohorts), and (2) studies with a larger number of patients in the IT phase. All study search, inclusion, and exclusion processes were performed by 2 independent researchers (H.A.L. and C.H.R.), and disagreements were resolved through mutual discussion.

This study is based on published data and did not use human materials and identifiable clinical data. Therefore, institutional board review was not indicated. Otherwise, the study was performed in accordance with the ethical guidelines of the 1975 declaration of Helsinki.

### Data items and collection process

Data collection was performed using a standardized form including (1) general information including author, affiliation, patient recruitment period, year of publication, and study design; (2) clinical information including the number of patients, sex, age, ALT, and HBV-DNA levels, HCC, mortality, phase change (eg, the overall number of cases, 5-year or 10-year estimation), and follow-up period; and (3) criteria defining the IT and IA phases. In the absence of numerical data, the estimated 5-year or 10-year occurrence rates were acquired from the descriptive graphs.^16^

### Quality assessment and risk of bias

Since possible candidate studies in the preliminary search were mostly observational studies, we used the Newcastle-Ottawa scale to assess the quality of the studies.^17^ Candidate studies in preliminary searches had similarly high scores in the compartments, including selection and exposure, except comparability. Studies with scores of 8–9 were regarded as having high quality, 6–7 as having medium quality, and those with scores of 5 or lower were considered low quality. Sensitivity analyses were performed excluding studies with the low quality following the recommendation that observational studies with a high risk of bias should be excluded from the review protocol.^14^

### Statistics

The principal summary measures were the pooled percentile rates of the clinical endpoints. The primary endpoint was the pooled incidence rate of HCC in the IT and IA cohorts, whereas the secondary endpoint was the pooled incidence rate of mortality in the IT and IA cohorts and phase change in the IT cohort. The random effects model was used considering that candidate studies were performed in different institutions and clinical heterogeneities among studies.^14^ Subgroup analysis was performed, including comparative series pooling odds ratios comparing the IT and IA cohorts regarding the pooled incidence rate of HCC. Since the random effects model averages the distribution of results affected by chance (ie, calculation of statistical heterogeneity is invalid), heterogeneity between results was shown by pooled estimates and 95% CI.^14^ Publication bias assessment was performed for analyses that included more than 10 cohorts using visual assessment of funnel plots and quantitative Egger test.^18^ If possible publication bias was noted (eg, 2-tailed *p*<0.1, Egger test), Duval and Tweedie’s^19^ trim and fill method was performed to calculate adjusted estimates. All statistical analyses were conducted using the Comprehensive Meta-Analysis version 3 (Biostat Inc., Englewood, NJ).

## RESULTS

### Study selection

**FIGURE 1:**
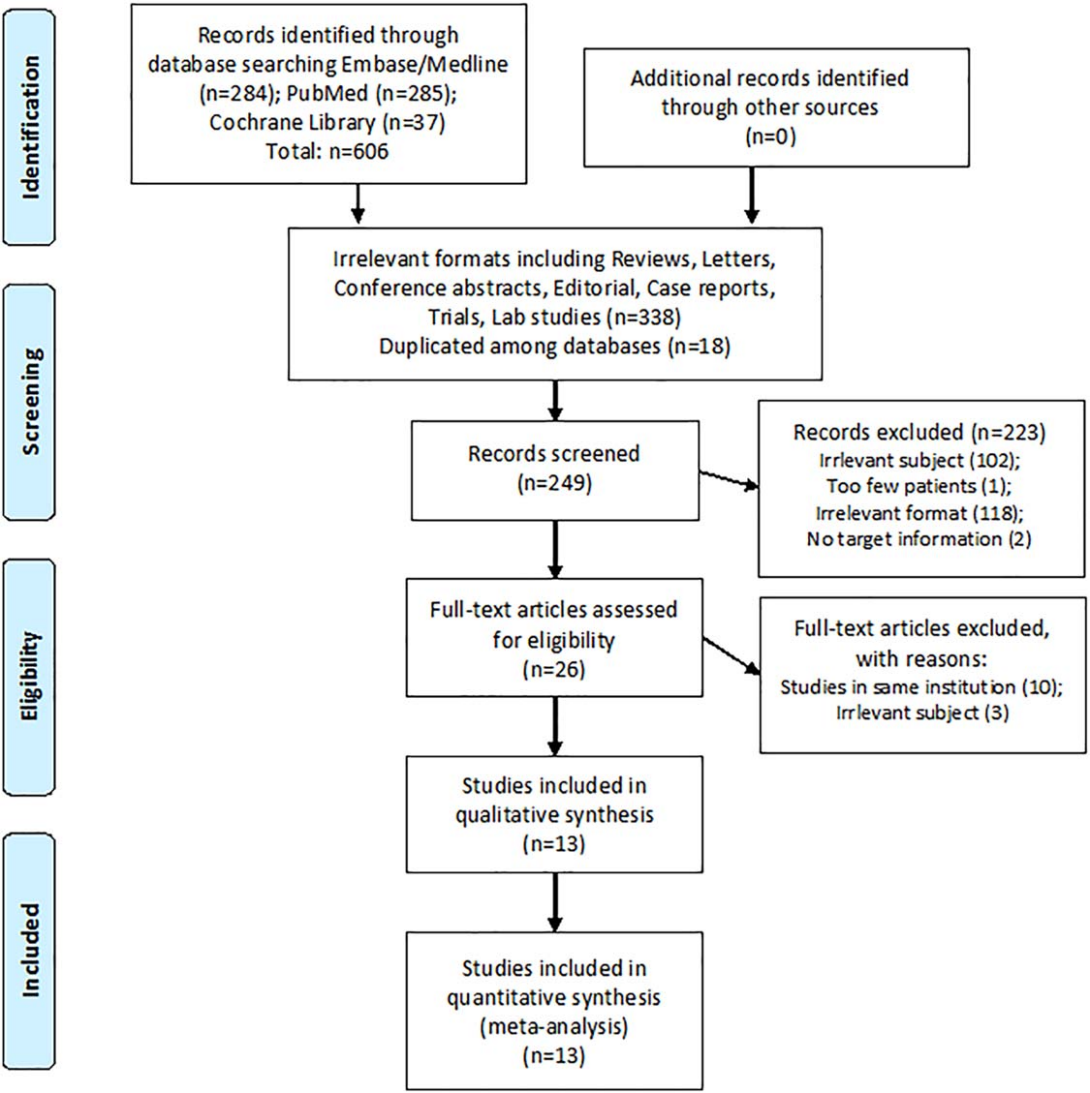
Study inclusion plot.

In the initial search across the database, 606 studies were identified. Among them, 356 studies were machine-filtered for irrelevant formats, such as reviews, letters, editorials, case reports, or duplication among databases. After filtering the abstracts and citations of 249 studies, 26 studies underwent full-text review. A full-text review was performed to identify the studies that met the inclusion criteria. Finally, 13 studies with 11,903 patients were included. The study inclusion process is summarized in Figure 1. Among the 13 included studies, 5 were comparative studies between IT and IA cohorts, whereas 8 were single-arm studies that recruited patients in the IT phase.

### Quality assessment and risk of bias

In the quantitative quality assessment, 3 comparative studies achieved 9 points (full points), and 2 studies achieved 8 points due to comparability (provision of a single clinical endpoint) and representativeness (small number of patients); therefore, all comparative studies were regarded to be of high quality. Single-arm studies achieved 7 points because they fulfilled all criteria other than comparability. All studies were included in the pooled analyses because no low-quality studies were found. The detailed scoring sheet is provided in Supplementary Table 1, http://links.lww.com/HC9/A37.

### Clinical characteristics of included studies

The clinical information and definitions of IT and IA phase of the included studies is shown in Table 1. In all the included studies, the overall median of median follow-up period was 62.4 months (range: 24.0–103.0 mo). The median of median age was 40.0 years (range: 29.0–53.5 y), and the prevalence of male sex was 59.6% (range: 31.8%–68.9%). The median of median ALT and HBV-DNA level was 39.5 IU/L (range: 19.0–156.0 IU/L) and 7.7 log_10_ IU/mL (range: 2.7–9.81 log_10_ IU/mL), respectively.

**TABLE 1:** Characteristics of included studies

References	Data source, country	Enrollment	Group	Patients, n	Male, n (%)	Age, years	Definition of phase	ALT, IU/L	HBV-DNA, log_10_ IU/mL	Follow-up time, months	HCC	*p*	Mortality	Phase change
Lee et al^20^	8 academic teaching hospitals, Korea	Jan 1989–Dec 2017	IT phase	946	429 (45.3)	36.8 (27.6–45.7)	HBV-DNA >20,000 IU/mL and ALT ≤40 IU/L	24.6 (19.0–32.0)	8.50 (7.45–8.23)	63.6	Overall: 10 cases (1.1%) 0.3% at 5 y 1.7% at 10 y	—	5 cases (0.5%) at 10 y	43.6% at 5 y 70.7% at 10 y
Kim et al^11^	Asan Medical Center	Jan 2000–Dec 2013	IT phase	413	276 (66.8)	38±11	HBV-DNA >20,000 IU/mL and ALT <ULN (AASLD)^a^	19 (16–25)	8.0 (7.0–8.4)	58.8 (28.8–103.2)	Overall: 24 cases (5.8%) 4.2% at 5 y 12.7% at 10 y HR 2.23 (95% CI 1.38–3.61)	*p*=0.001	1.9% at 5 y 9.7% at 10 y HR 2.73; 95% CI 1.54–4.84	—
			IA phase	1497	973 (65.0)	40±11	HBV-DNA >20,000 IU/mL and ALT >2×ULN (AASLD)^a^	156 (95–308)	7.7 (6.9–8.3)	80.4 (44.4–123.6)	Overall: 54 cases (3.6%) 1.6% at 5 y 6.1% at 10 y		0.8% at 5 y 3.4% at 10 y	—
Hui et al^21^	Nethersole Hospital, China	Jan 1997–Dec 1998	IT phase	57	34 (59.6%)	31 (18–41)	HBV-DNA >10^7^ copies/mL, ALT <7–53 U/L for men and <7–31 U/L for women on 3 consecutive readings 6 mo apart before the initial liver biopsy	30 (4–42)	9.81 (7.12–10.00)	60	0.0% at 5 y	—	0.0% at 5 y	57 cases (15.8%) at 5 y
Lee et al^22^	Yonsei University Severance Hospital and Cha Bundang Medical Center in Korea, Prince of Wales Hospital in China	Jan 2010–Dec 2016	IT phase	194	84 (43.3)	31.6±6.1	Age <40 y, HBV-DNA >6 log_10_ IU/mL, and persistently normal ALT level (≤40 IU/L) during the follow-up	25.0 (19.0–32.0)	8.1±0.6	62.1 (41.8–86.1)	0.0% at 5 y 0.0% at 9 y	—	0.0% at 5 y 0.0% at 9 y	Overall: 97 cases (50.0%) 34.6% at 5 y 52.7% at 9 y
			IA phase	454	313 (68.9)	42.8±11.5	Who meet the treatment guidelines and reimbursement criteria for NA therapy in each country	142.5 (44.5−39.8)	4.0±1.7		4 cases (0.9%) 0.7% at 5 y 1.35% at 9 y	—	0.0% at 5 y 0.0% at 9 y	—
Lee et al^23^	Yonsei University Severance Hospital	Jan 2006–Dec 2012	IT phase	126	62 (49.2)	47.7±11.1	HBV-DNA level of ≥20,000 IU/mL and persistently normal ALT level (≤40 IU/L) during the follow-up	23.4±7.8	6.9±2.0	96.6	1.1% at 5 y 2.7% at 10 y HR 2.327 (95% CI 0.475–11.39)	—	3.0% at 5 y 4.6% at 10 y HR 1.341 (95% CI 0.457–3.933)	—
			IA phase (with viral response)	641	409 (63.8)	53.5±10.7	Who meet the treatment guidelines and reimbursement criteria for NA therapy in Korea	24.9±10.2	2.7±0.9		1.0% at 5 y 2.9% at 10 y	–	2.6% at 5 y 6.1% at 10 y	—
Jang et al^24^	16 university-affiliated hospitals, Korea	Jan 2007–Dec 2018	IA phase	4492	2680 (59.7)	47.1±11.9	Who met the AASLD guidelines for NA treatment	102 (60–200)	7.2 (6.0–8.2)	61.2 (39.6–82.8)	0.2% at 2 y 0.7% at 5 y 2.1% at 8 y	—	—	—
Nam et al^25^	Seoul National University Hospital, Korea	Jan 2007–June 2013	IA phase	325	192 (59.1)	43.8±12.1	positive HBeAg at the time of antiviral therapy initiation	46.9±40.2	7.17±1.2	—	17 cases (5.2)	—	—	—
Seong et al^26^	Samsung Medical Center, Korea	Jul 1998–Dec 2006	IT phase	301	189 (62.8)	35 (25–44)	ALT <35 U/L for males and 25 U/L for females HBV-DNA >7 log IU/ml	22 (16.5–27.5)	8.10 (7.83–8.28)	62.4 (12.0–213.6)	0.5% at 5 y 4.3% at 10 y	—	—	—
Yapali et al^27^	Liver clinics at the University of Michigan Health System, USA	Jan 1999–Jan 2010	IT phase	24	14 (31.8)	29 (18–45)	HBV-DNA >20,000 IU/mL and ALT< 40 IU/L	37±17	—	51 (12–164)	0 cases (0.0%)	—	—	Overall 6 cases (25%) 55% at 5 y
			IA phase	20			HBV-DNA >20,000 IU/mL and ALT≥40 IU/L		—		0 cases (0.0%)	—	—	—
Behera et al^28^	Institute of Medical Sciences & SUM Hospital, India	Mar 2015–Aug 2017	IA phase	78	TDF: 19 (50) ETV: 24 (60)	TDF: 43.6 ETV 37.8	HBV-DNA >2×10^5^ IU/ml ALT >2×ULN*	TDF: 92±46.8 ETV: 122.67±77	TDF: 6.24±1.03 ETV: 6.11±0.7	24.0	0.0% at 2 y	—	0 cases	—
Lee et al,^22^ unpublished data^b^	Korea University Hospital, Yonsei University Severance Hospital	2007–2016	IA phase	928	552 (59.5)	42.1 (33.8–51.5)	Who meet the treatment guidelines and reimbursement criteria for NA therapy	109.0 (73.0–204.0)	8.03 (6.97–8.23)	87.4 (62.1–114.1)	Overall: 36 cases (3.9%) 1.4% at 5 y 5.4% at 10 y	—	—	—
Kwon et al^29^	Five Catholic university St. Mary’s Hospital	—	IT phase	522	—	36	HBV-DNA >1,000,000 IU/ml, ALT <80 IU/L	—	—	75	0.3% at 5 y 1.3% at 10 y	0.46	—	43.6% at 5 y 73.6% at 10 y
		—	IA phase	609	—	41	HBV-DNA >1,000,000 IU/ml, ALT >80 IU/L	—	—	61	0.9% at 5 y 3.0% at 10 y		—	—
Yoo et al^12^	Three tertiary hospitals, Korea	1994–2017	IT phase	276	—	42.5±12.4	High HBV-DNA levels, ALT <80 IU/mL	42 (31–56)	8.26 (7.20–8.93)	103 (51–145)	17 cases (6.2%)	—	—	—

Variables are expressed as mean±SD, median (interquartile range), or n (%).

^a^ The criteria of the AASLD, American Association for the Study of Liver Diseases: <19 U/L for females and <30 U/L for males.

^b^ Presented in the Liver week 2021, virtual conference.

Abbreviations: ALT indicates alanine aminotransferase; ETV, entecavir; ; IA, immune-active; IT, immune-tolerant; NA, nucleos(t)ide analog; TDF, tenofovir disoproxil fumarate.

The median prevalence of male sex was 49.2% (range: 31.8%–66.8%) in the IT cohort and 59.9% (range: 50.0%–68.9%) in the IA cohort. The median of median age in IT cohort was lower than that of IA cohort (36.0 y, range: 29–47.7 y vs. 42.8 y, range: 37.8–53.5 y). The median of median ALT level in IT cohort was lower than that of IA cohort (24.8 IU/L, range: 19–42 IU/L vs. 105.5 IU/L, range: 24.9–156 IU/L). The median of median HBV-DNA levels in IT cohort was higher than that of IA cohort (8.1 log_10_ IU/mL, range: 6.9–9.8 log_10_ IU/mL vs. 6.7 log_10_ IU/mL, range: 2.7–8.0 log_10_ IU/mL).

### Primary outcome

In all included studies, the median 5-year pooled incidence rate of HCC was 0.5% (range: 0.0%–6.2%) in the IT cohort and 0.9% (range: 0.0%–5.2%) in the IA cohort.

Among the 5 comparative studies, the study by Kim et al^11^ reported a significantly higher 5-year cumulative incidence rate of HCC in the IT cohort than that in the IA cohort (4.2% vs. 1.6%; *p*=0.001), whereas 3 studies demonstrated statistically similar 5-year cumulative incidence rates of HCC between the 2 groups (0.0% vs. 0.7% in the study by Lee et al^22^ 1.1% vs. 1.0% in the study by Lee et al^23^ and 0.3% vs. 0.9% in the study by Kwon et al^29^).^11^,^29^ The last study by Yapali et al^27^ reported a 0% cumulative incidence rate of HCC in both cohorts.

**FIGURE 2:**
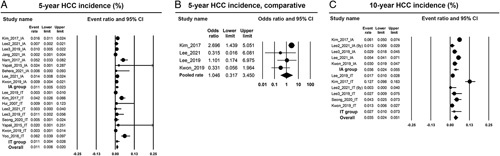
Forest plots of pooled analyses of the 5-year incidence rate of HCC (A), odds ratio of the 5-year incidence rate of HCC (B), and the 10-year incidence rate of HCC (C). Abbreviations: IA indicates immune-active; IT, immune-tolerant.

The pooled results of the clinical outcomes are presented in Table 2. The pooled 5-year pooled incidence rate of HCC was 1.1% (95% CI: 0.6%–2.0%) in all included studies. The pooled 5-year incidence rate of HCC was statistically similar between IT and IA cohorts (1.1%, 95% CI: 0.4%–2.8% vs. 1.1%, 95% CI: 0.5%–2.3%; *p* for difference=0.976) (Figure 2A). The pooled 5-year odds ratio of HCC risk in the comparative series was 1.05 (95% CI: 0.32–3.45; *p*=0.941) (Figure 2B). The pooled 10-year incidence rate of HCC was 3.5% (95% CI: 2.4%–5.1%) in all included cohorts. The pooled 10-year incidence rate of HCC was statistically similar between the IT and IA cohorts (2.7%, 95% CI: 1.0%–7.3% vs. 3.6%, 95% CI: 2.4%–5.5%, *p* for difference=0.587) (Figure 2C).

**TABLE 2:** Pooled results of clinical endpoints

Subjects	Cohort (n)	Patients (n)	Effect size (%) (95% CI)	Subgroup *p*	Eggers’ *p*	Trimmed value (%)
5-year HCC rate						
All cohorts	18	11,903	1.1 (0.6–2)		0.082	1.90
IT cohorts	9	2859	1.1 (0.4–2.8)	0.976		
IA cohorts	9	9044	1.1 (0.5–2.3)			
Comparative series	4^a^	3201	OR: 1.05 (0.32–3.45, *p*=0.941)			
10-Year HCC rate						
All cohorts	11	6651	3.5 (2.4–5.1)		0.019	3.80
IT cohorts	6	2502	2.7 (1.0–7.3)	0.587		
IA cohorts	5	4129	3.6 (2.4–5.5)			
5-Year mortality rate						
All cohorts	8	3460	1.7 (0.1–2.8)		NA (<10 cohorts)	
IT cohorts	4	790	1.9 (1.1–3.4)	0.285		
IA cohorts	4	2670	1.0 (0.3–2.9)			
5-year phase change rate						
IT cohorts	5	1743	36.1% (29.5–43.2)			

^a^ The study by Yapali and colleagues was not included because they reported 0% in both arms.

Abbreviations: IT indicates immune-tolerant; IA, immune-active.

### Secondary outcomes

**FIGURE 3:**
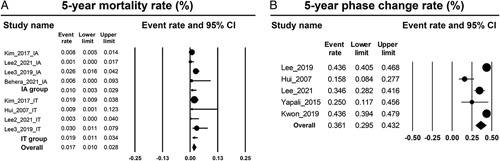
Forest plots of pooled analyses of the 5-year incidence rate of mortality (A), and the 5-year incidence rate of phase change in the IT cohort (B). Abbreviations: IA indicates immune-active; IT, immune-tolerant.

The 5-year pooled incidence rate of mortality ranged from 0.0% to 3.0% in the IT cohort and from 0.0% to 2.6% in the IA cohort. The pooled 5-year incidence rate of mortality was statistically similar between the IT and IA cohorts (1.9%, 95% CI: 1.1%–3.4% vs. 1.0%, 95% CI: 0.3%–2.9%, *p* for difference=0.285) (Figure 3A).

Five studies reported the 5-year cumulative incidence rate of phase change from the IT to IA phase in the IT cohort, ranging from 15.8% to 43.6%. The pooled 5-year incidence rate of phase change was 36.1% (95% CI: 29.5%–43.2%) (Figure 3B).

### Publication bias

A possible publication bias was noted in the pooled analyses of the 5-year and 10-year incidence rate (Egger test, *p*=0.082 and Egger test, *p*=0.019, respectively). The trimmed values obtained using Duval and Tweedie’s method were slightly higher than the untrimmed values. The quantitative results of the assessment of publication bias are presented in Table 2. The funnel plots are shown in Supplementary Figure 1, http://links.lww.com/HC9/A37.

## DISCUSSION

To date, it has been accepted by international guidelines that CHB patients in the IT phase are at negligible risk of disease progression.^6^,^7^,^21^ However, this concept has recently been challenged by recent studies,^11^,^12^ and international guidelines recently endorsed that AVT may be considered for HBeAg-positive patients aged >30 or 40 years with a high HBV-DNA level and normal ALT level, despite the low level of evidence.^6^,^7^ In this meta-analysis, we identified that the pooled incidence rates of HCC and mortality were low in the IT phase and comparable with those in the IA phase in noncirrhotic HBeAg-positive CHB patients. In addition, the pooled incidence rate of phase changes in the IT cohort was high.

Our study has several clinical implications. First, to the best of our knowledge, this is the first meta-analysis to include 11,903 patients from 13 studies with more than 5-year follow-up, which compared the long-term prognosis between untreated IT cohort and IA cohort treated with AVT. Studies that recruited patients with liver cirrhosis were excluded because liver cirrhosis is the single most potent risk factor for HCC or mortality, and patients with liver cirrhosis should not be allocated to the IT phase.^9^,^10^,^15^,^16^ Finally, we found no significant differences in the pooled 5-year and 10-year incidence rate of HCC (1.1% vs. 1.1% and 2.7% vs. 3.6%, respectively), and the pooled odds ratio of the 5-year incidence rate of HCC between IT and IA cohorts was 1.05. In addition, the pooled 5-year incidence rate of mortality was statistically comparable between the IT and IA cohorts (1.9% vs. 1.0%).

Second, the pooled 5-year and 10-year incidence rates of HCC in the IT cohort were low (1.1% and 2.7%, respectively). A recent study by Kim et al^11^ showed high HCC incidence rates of 4.2% and 12.7% at 5 and 10 years, respectively, in the IT cohort. However, other studies reported an extremely low risk of HCC, ranging 0.0%–1.1% at 5 years and 0.0%–4.3% at 10 years.^20^–^23^,^26^,^27^,^29^ In addition, our study showed that the pooled 5-year pooled incidence rate of mortality was only 1.9% in the IT cohort. In the literature, except for the study by Kim and colleagues, most studies have shown a low 10-year pooled incidence rate of mortality (0.0%–4.6%).^11^,^20^–^23^ These findings indicate that AVT may not be required in CHB patients in the IT phase, as they have favorable long-term outcomes.

Third, our study showed that noncirrhotic patients in the IA phase treated with AVT also have low risks of HCC or mortality, similar to those of patients in the untreated IT phase. In our study, the pooled 5-year and 10-year incidence rates of HCC were 1.1% and 3.6%, respectively, and the 5-year mortality rate was 1.0% in the IA cohort. In 5 comparative studies, the pooled odds ratio of the 5-year incidence rate of HCC was 1.05 (*p*=0.941). Only one study by Kim et al^11^ suggested that the cumulative incidence rate of HCC in patients treated with IA was even lower than that in untreated IT patients. The reason why the cumulative incidence rates of HCC and mortality in the IT cohort vary in previous studies^11^,^20^–^30^ could be that how strictly defining the true IT phase by eliminating subjects who are at high risks, such as having significant liver fibrosis. Although the number of comparative studies is insufficient to draw firm conclusions, our results suggest that the disease course of noncirrhotic IA phase patients can become favorable with potent AVT, similar to that of IT phase patients.

Fourth, the definition of the IT phase based on clinical parameters without histological assessment has been debated. The median HBV-DNA level of the IT cohort was higher than that of the IA cohort (8.1 vs. 6.7 log_10_ IU/mL). Among all included studies, the highest cumulative incidence rate of HCC in the IT cohort was reported in a study by Kim et al,^11^ where the IT phase was defined as an HBV-DNA level ≥20,000 IU/mL. However, other studies with more stringent criteria of higher HBV-DNA levels (>10^7^ copies/mL in Hui and colleagues, >10^6^ IU/mL in Lee and colleagues and Kwon and colleagues, and >10^7^ IU/mL in Seong and colleagues) reported a lower 5-year and 10-year cumulative incidence rate of HCC (0.0%–0.5% and 1.3%–4.3%, respectively).^21^,^22^,^26^,^29^ These findings suggest that higher levels of HBV-DNA should be used to define the true IT phase with favorable long-term outcomes, which was supported by a recent study by Kim et al^30^ showing the highest HCC risk in patients with HBV-DNA levels of 6–7 log_10_ IU/mL compared to the lowest risk in those with >8 log_10_ IU/mL.

Fifth, 2 studies in our meta-analysis excluded subjects with advanced fibrosis or liver cirrhosis.^20^,^21^ A recent study by Lee et al^20^ excluded patients with or suspected to have significant fibrosis, based on the histological or clinical use of noninvasive surrogates, resulting in an extremely low 5-year and 10-year cumulative incidence rate of HCC (1.1% and 1.7%, respectively). In other study by Hui et al,^21^ after excluding patients with fibrosis stage >1 on initial liver biopsy, 5-year cumulative incidence rate of HCC was 0.0%. In contrast, another study which did not stringently exclude patients with significant or advanced fibrosis showed high incidence rate of HCC in the IT cohort (4.2% at 5-year and 12.7% at 10-year, respectively).^11^ Although several studies have excluded subjects with liver cirrhosis, those with significant liver fibrosis might have been misclassified into the IT phase, resulting in poor outcomes in the IT cohort.^12^,^20^–^23^,^26^,^27^,^29^ As fibrosis is the single most important predictor of clinical outcomes, detailed assessment of fibrotic burden and the corresponding exclusion of subjects with significant fibrosis are strongly required to define the true IT phase. Interestingly, a previous study showed that liver fibrosis progression is uncommon in IT phase and IA phase with AVT and the risk of liver fibrosis progression was comparable between the 2 groups.^31^

Finally, it is unclear whether age should be considered when defining the IT phase. In our meta-analysis, the median age of the IT cohort was lesser than that of the IA cohort (36.0 vs. 42.8 y). Because all studies in this meta-analysis excluded patients with other cause of chronic liver injury such as NAFLD or significant alcohol use, the reason why untreated young patients developed HCC might be explained in part by the HBV genome integration into host.^32^,^33^ Although most studies did not define the IT phase using age limits, studies including patients younger than 40 years reported no HCC incidence at 5 years,^21^,^22^ and other studies reported that HCC only developed in patients older than 40 years in HBeAg-positive patients.^20^–^22^,^26^ However, as patients get older during the disease course, the 1-time use of age factors to define the IT phase might be inappropriate. Our study showed that the pooled 5-year cumulative incidence rate of phase change from IT to IA requiring AVT was not negligible (36.1%). When considering the age gap between the IT and IA cohorts of ~6 years in our study, serial follow-up of patients in the IT phase and proper initiation of AVT should be emphasized.

We are also aware of several unresolved issues. First, a meta-analysis of observational studies has potential pitfalls because uncontrolled confounders may affect the pooled estimates.^14^ It might be difficult to design a study randomizing a sufficiently large enough number of population to find small differences. Therefore, a meta-analysis of observational studies is one of the few available options to support clinical decisions. In addition, readers might consider the pooled estimates and its CIs because we used a random effects model, which estimates the mean of a distribution of effects. Second, the diagnostic criteria for the IT phase have not been unified among studies, primarily owing to the lack of a diagnostic consensus. However, we compared the risks of clinical outcomes according to each diagnostic criterion, suggesting stringent diagnostic criteria for the IT phase. Current guidelines recommend that AVT can be considered in patients older than 30 or 40 years, in spite that they are considered as in IT phase.^20^ Because our meta-analysis could not provide additional results in the subgroup of younger patients <30 or 40, further studies with stringent age criteria might be required to reveal the clinical implication of IT phase. Third, the definitions of liver cirrhosis were different among the studies. Two studies used liver stiffness value, assessed using transient elastography and fibrosis-4 index, and another study used AST-to-platelet ratio index score to diagnose liver cirrhosis.^20^ Three studies used liver biopsy to diagnose liver cirrhosis.^12^,^21^,^27^ In other studies, liver cirrhosis was diagnosed according to clinical criteria based on ultrasonographic findings, clinical features of portal hypertension, and thrombocytopenia.^11^,^22^–^26^,^28^,^29^ Because of this potentially biased exclusion of patients with cirrhosis, our results should be interpreted with cautions. Fourth, the prognosis of CHB patients could be affected by various clinical factors such as age, sex, and diabetes, therefore, sensitivity analysis according to these factors might be clinically relevant.^34^,^35^ However, because most studies included in our meta-analysis did not provide clinical outcomes in specific subgroups, various sensitivity analysis was not feasible. Fifth, to make certain the definition of the IT phase, comparing patients who remained in IT phase and who transit to IA phase would be more appropriate. However, this analysis was not feasible, because most of include studies lack those data. Finally, most observational studies focused on East Asian patients, selection bias may limit the generalizability of the results to other populations, particularly Caucasians.

In conclusion, the pooled incidence rates of HCC and mortality were comparable between untreated IT and treated IA phases in noncirrhotic HBeAg-positive CHB patients in this meta-analysis, supporting the timely initiation of AVT during the disease course of CHB. In addition, we found that the pooled incidence of a phase change from IT to IA requiring AVT was not negligible, which might necessitate close monitoring during the IT phase. Further studies are warranted to define the more stringent criteria for the IT phase.

## Supplementary Material
