# Supplementary material for: Comparable outcomes between immune-tolerant and active phases in noncirrhotic chronic hepatitis B: a meta-analysis
Source: Hepatol Commun. 2023 Jan 18;7(2):e0011. doi: 10.1097/HC9.0000000000000011 (PMC9851695; doi:10.1097/HC9.0000000000000011)
Supplement: Supplementary file 1 [file hc9-7-e0011-s001.docx]

**Supplement 1.** Draft of search strategy

**Search strategy in Embase and Medline**

Embase search:

#1 ('immune tolerant' OR 'immune tolerance'/exp OR 'immune tolerance') AND hcc

#2 #1 NOT ('editorial'/it OR 'review'/it)

*#1 is our basic strategy using Emtree; #2 is to filter studies with irrelevant formats (e.g. reviews, editorials, letters, conference abstracts) or not relevant clinical studies (e.g. case reports, in vivo studies)

**Search strategy in Pubmed and Cochrane library**

Search term: ('immune tolerant' OR 'immune tolerance'/exp OR 'immune tolerance') AND hcc

Using search query, filters to include clinical trial (I~IV), comparative study, clinical study, controlled clinical trial, multicenter study, and observational study were used. We did not use any filter in Cochrane library.

| **Supplement Table 1**. Scoring sheet according to New-Castle Ottawa scale | | | | |  |  |  |  |  |
| --- | --- | --- | --- | --- | --- | --- | --- | --- | --- |
|  | Selection | | | | Comparability | Outcome | | | **Overall score  (9 to be full )** |
|  | 1 | 2 | 3 | 4 | 1 | 1 | 2 | 3 |  |
|  | Representativeness of the exposed cohort | Selection of the non- exposed cohort | Ascertainment of exposure | Outcome of interest was not present at start of study | Comparability of cohorts on the basis of the design or analysis | Assessment of outcome | Was follow-up long enough for outcomes to occur | Adequacy of follow up of cohorts |  |
| Lee, 2019 (multi-institution) | 1 | 1 | 1 | 1 | 0 | 1 | 1 | 1 | 7 |
| Kim, 2017 | 1 | 1 | 1 | 1 | 1 | 1 | 1 | 1 | 8 |
| Hui, 2007 | 1 | 1 | 1 | 1 | 0 | 1 | 1 | 1 | 7 |
| Lee, 2021 | 1 | 1 | 1 | 1 | 1 | 1 | 1 | 1 | 8 |
| Lee, 2019 | 1 | 1 | 1 | 1 | 1 | 1 | 1 | 1 | 8 |
| Jang, 2021 | 1 | 1 | 1 | 1 | 0 | 1 | 1 | 1 | 7 |
| Nam, 2017 | 1 | 1 | 1 | 1 | 0 | 1 | 1 | 1 | 7 |
| Seong, 2020 | 1 | 1 | 1 | 1 | 0 | 1 | 1 | 1 | 7 |
| Yapali, 2015 | 0 | 1 | 1 | 1 | 1 | 1 | 1 | 1 | 8 |
| Behera, 2021 | 1 | 1 | 1 | 1 | 0 | 1 | 1 | 1 | 7 |
| Lee, 2021 | 1 | 1 | 1 | 1 | 0 | 1 | 1 | 1 | 7 |
| Kwon, 2019 | 1 | 1 | 1 | 1 | 1 | 1 | 1 | 1 | 8 |
| Yoo, 2018 | 1 | 1 | 1 | 1 | 0 | 1 | 1 | 1 | 7 |

**Supplement Figure 1.**

1. Funnel plot of the cumulative incidence rate of hepatocellular carcinoma at 5-year

1. Funnel plot of the cumulative incidence rate of hepatocellular carcinoma at 10-year
